# Environmental history determines forest habitat network functionality: The need for landscape planning in Sweden

**DOI:** 10.1007/s13280-026-02353-7

**Published:** 2026-03-01

**Authors:** Per Angelstam, Taras Yamelynets, Michael Manton, Jakub Bubnicki, Bengt-Gunnar Jonsson, Grzegorz Mikusinski, Johan Svensson, Lucas Dawson

**Affiliations:** 1 Department of Forestry and Wildlife Management, University of Inland Norway, Anne Evenstads Vei 80, 2480 Koppang, Norway; 2 Faculty of Geography, Ivan Franko National University of Lviv, Doroshenko Street 41, Lviv, 79000 Ukraine; 3 Bioeconomy Research Institute, Vytautas Magnus University, Studentu G. 13, 53362 Akademija, Kauno r., Lithuania; 4 Mammal Research Institute Polish Academy of Sciences, Stoczek 1, Białowieża, Poland; 5 Department of Natural Sciences, Mid Sweden University, 851 70 Sundsvall, Sweden; 6 School for Forest Management, Swedish University of Agricultural Sciences, PO Box 43, 739 21 Skinnskatteberg, Sweden; 7 Department of Wildlife, Fish and Environmental Studies, Faculty of Forest Sciences, Swedish University of Agricultural Sciences, 90183 Umeå, Sweden

**Keywords:** Focal species, Green infrastructure, Habitat loss thresholds, High conservation value forests, Nature restoration, Spatial planning

## Abstract

Harvesting naturally dynamic forests causes losses of habitat quality and functional connectivity. Focusing on Sweden as a case study of high-yield rotation forestry, we provide analyses supporting spatial prioritisation of protection, management and restoration of representative functional forest habitat networks. Habitat suitability index modelling of focal bird species was used to analyse how forest naturalness, habitat patch size and functional connectivity affect representative forest habitat networks in Sweden’s five ecoregions. Habitat modelling for the least demanding bird species showed that of the mountain ecoregion 57–77% was functional, but in the other three boreal ecoregions only 8–9% were functional. For nemoral forests, the proportions of functional habitat networks were < 3%. More demanding species have even less functional habitat. We highlight the importance of the mountain ecoregion for forest biodiversity conservation, and the urgent need for landscape planning of protection, conservation management and nature restoration in Sweden.

**Supplementary Information:**

The online version contains supplementary material available at 10.1007/s13280-026-02353-7.

## Introduction

Forest landscapes are expected to satisfy a growing demand for wood, biomass and socio-cultural values, while simultaneously conserving forest biodiversity in terms of species, habitat and processes, as well as supporting climate change mitigation and adaptation (e.g. European Commission 2020, 2021; CBD 2022; European Commission 2023). Stressing forests’ multifunctional role, both integrated (Kraus and Krumm 2013) (land sharing) and segregated (Seymour and Hunter 1992) approaches (land sparing) to satisfy different Sustainable Forest Management (SFM) policy objectives (European Commission 2021) have been proposed (Himes et al. 2022). However, evidence suggests that a segregated forest landscape management approach is most effective for pursuing multiple objectives at the landscape level (e.g. Nagel et al. 2025). In-depth case studies in Nova Scotia (Canada) (Himes et al. 2022), Finland (Blattert et al. 2023) and Oregon (USA) (Mast et al. 2025) indicate that a triad approach offers a promising solution. This involves a landscape-level mix of intensively managed forests aimed at biomass production, areas providing multiple services and functions such as carbon storage, and sufficient protected areas and set-asides for conservation that together form representative and functionally connected habitat networks. Such a triad approach thus provides opportunities for multiple value chains that encompass direct use, indirect use, bequest and existence values (Jonsson et al. 2019). Realising this calls for a place-based holistic landscape approach to spatial planning that integrates social and ecological systems (IPBES 2024). However, the real world includes a wide range of wicked challenges including climate change and a diversity of conflicting objectives, actors and stakeholder with unequal power and opportunities (Nikolakis and Innes 2020).

Gradual transformation of naturally dynamic forest landscapes to cropping systems has a history of being replicated globally (e.g. Thomas 1956; Angelstam et al. 2021). Williams (2003, 146) highlighted two “theatres of action” based on the connection between demand and supply, which are linked by flow of wood using seas and other waterways, and later by expanding frontiers of infrastructure development supporting forest use. A prime example is the industrial revolution in Western Europe, which triggered wood mining in intact forest landscapes in Eastern Europe (Naumov et al. 2018). Thus, the areas of remnant forests with higher levels of naturalness are shrinking, unless inaccessible remote locations or rough terrain offers protection (e.g. Potapov et al. 2017; Naumov et al. 2018; Svensson et al. 2019). Subsequently, even-aged silviculture developed in Europe (Angelstam et al. 2022) as a base for effective cropping systems for sustained yield of wood for forest-based industries.

Consequently, exploitation of naturally dynamic forests forms expanding frontiers of habitat loss, and simultaneously also frontiers of establishing protected areas aiming at biodiversity conservation. Angelstam et al. (2021) explored net effects of these frontiers in 16 case study regions globally. They concluded that despite evidence-based conservation targets, and awareness of the need for spatial segregation of conservation and use (e.g. Nagel et al. 2025), the net trajectories for biodiversity conservation were generally negative. In the European Union Member States, the vast majority (84%) of the assessments of the 69 forest habitat types at the EU level have an unfavourable conservation status, and the boreal forests have the worst status (100% unfavourable) (European Commission 2022). In particular, north European boreal countries like Sweden illustrate this (Ahlström et al. 2022; Angelstam and Manton 2021). Conversely, this coincides with a strong focus on high sustained yield forestry.

The successful development of intensive even-aged rotation forestry in Sweden is illustrated by that while the country hosts only 0.7% of the world’s and 16% of EU’s forest area, yet the combined total export of sawn soft-wood, pulp, paper, and board is the fourth largest in the world after Canada, Russia and USA (Naumburg 2024). Challenged with the unrealistic desire to achieve “more of everything” from forests in landscapes with a wide range of forest owners (Beland Lindahl et al. 2017; Felton et al. 2020; Angelstam et al. 2023), Sweden is thus a good case study of attempts to sustain biodiversity and multifunctional landscapes in the context of high-yield wood production.

Wood production and environmental considerations were set as equal objectives of Swedish forest policy in 1993, and this ambition is still upheld (Direktiv 2024). Evidence-based conservation biology knowledge supports Hanski’s (2011) “third-of-third approach” with a third of the land area managed as multi-use conservation landscapes, within which a third of the area is protected. Consistent with this, the CBD (2022) and the EU Biodiversity Strategy for 2030 (European Commission 2020) have set out the aim to allocate at least 30% of the land areas for biodiversity conservation and to effectively manage them. In the EU, one-third should be strictly protected (including all primary and old-growth forests) and the rest be conserved, for example, by using “closer-to-nature” methods (European Commission 2023).

Comprehensive assessments of the implementation of environmental dimensions of SFM policy on the ground in Sweden show, however, that desired consequences have failed to materialise (Angelstam and Andersson 2001; Skogsstyrelsen 2022). Key problematic challenges include conservation of viable populations of naturally occurring species and their habitats, ecosystem integrity and resilience, and social values (Angelstam et al. 2023). Formally protected areas, voluntary set-asides, low-productive forests, and nature considerations in forestry aim at resolving these challenges (Angelstam et al. 2020; SCB 2025). Currently, formally protected areas on productive land contribute most in terms of habitat quality (Kyaschenko et al. 2022). Protected areas cover 6.3% of Sweden’s productive forest land area, and 9.4% of all forest land including both productive and unproductive forests is protected (SCB 2025, 21). However, there is a stark contrast between the proportion of formally protected areas in the remote mountain forests in north-westernmost Sweden (59.1%), and the rest of the country where wood production dominates (3.0–5.8% formally protected) (SCB 2025, 29).

Thus, the implementation of environmental dimensions of SFM policy in Sweden exposes a gap between forest and conservation policy on the one hand, and reality on the other. This gap calls for protection of the few remaining high conservation value forests (HCVF), landscape-scale planning, appropriate conservation management regimes and major investments in nature restoration (European Commission 2020; Larsen et al. 2022). In turn, this requires spatial prioritisation to expand functionally connected representative habitat networks as a green infrastructure (Svensson et al. 2019, 2022). A major challenge is to develop simple and robust approaches to assess the state of habitat networks and to visualise for spatial planners the amount and location of representative and functional habitat clusters and hotspots (e.g. Wang et al. 2025). Additionally, approaches aiming at forest ecosystem resilience including adaptative capacity to cope with climate change need to be addressed throughout the entire landscape (e.g. Mina et al. 2022).

Given clear evidence-based national and international conservation objectives and increasingly intensive forestry in Sweden, it is urgent to assess the extent to which networks of patches of different forest types and ecoregions are functional. This depends not only on the areas set aside for biodiversity conservation (e.g. Barnes et al. 2018). A key additional requirement is adequate habitat quality in terms of an acceptable level of naturalness of forests and woodlands (e.g. Winter 2012). In addition, sufficient patch size and functional connectivity at different spatial scales required by species with different life history traits need to be satisfied (e.g. Angelstam et al. 2020). A key prerequisite of the aforementioned study focusing on Sweden was access to field surveys of forests with high conservation values. An alternative approach to mapping of forests with high conservation value is to model levels of naturalness by analysing wall-to-wall biophysical variables capturing the level of naturalness of local forest areas (e.g. Assmann et al. 2025), and socio-economic drivers affecting naturalness (Sanderson et al. 2002). Bubnicki’s et al. (2024) recent development of a model that predicts wall-to-wall the relative likelihood of forests that represent different levels of forest naturalness in Sweden is thus indeed timely. Trained on the Swedish national database of HCVFs surveyed in the field, and with a spatial resolution of 1 ha, it predicts the occurrence of forests representing the full gradient from degraded forests with low levels of naturalness suitable for intensive wood production to those with high biodiversity conservation values requiring protection. Bubnicki’s et al. (2024) modelling of forest naturalness thus fills an urgent gap as a contribution for assessing the achievement of evidence-based conservation targets, and supporting spatial landscape planning by attributing relative forest naturalness ranks across entire forest landscapes.

The aim of this study is to address five topics supporting wall-to-wall assessment of the functionality of representative forest types in Sweden as a base for prioritisation of sufficient amounts of areas for protection, conservation management and restoration, as well as for wood production. First, we map the spatial distribution and amount of the four dominating forest habitat network types comparing two levels of predicted naturalness rank in all five Swedish ecoregions. Second, we apply habitat suitability modelling to assess the functionality of habitat networks by applying knowledge about area requirements and sensitivity to habitat fragmentation of virtual species with different habitat demands. Third, we identify the ecoregional proportions and patch sizes of currently formally protected forests as well as forests with predicted high naturalness ranks. Fourth, we identify overlaps and gaps in the amount of officially mapped high conservation forests. Fifth, as an alternative to focus conservation on habitat networks, we also identify hotspot areas of different sizes matching different levels of ambition for biodiversity conservation, as well as land ownerships. We discuss challenges and opportunities for prioritising protection, management and nature restoration, as well as landscape planning among different forest owner categories.

## Materials and methods

### Sweden’s forests as a case study

Following the terminology of Stake (1995), a case study is a “bounded” separate entity in terms of place and space with physical boundaries hosting a neighbourhood, particular planning and management organisations, and histories. With a single case-study approach, one can do in-depth exploration of a specific bounded system, e.g. Blicharska et al. (2020) about the Bialowieza massif in Poland, and Mast et al. (2025) about Oregon’s Coast Range in the USA. This study focuses on Sweden’s forests as a single case study about the challenges for biodiversity conservation within a world-leading high-yield rotation forestry system (see Angelstam et al. 2022).

Forests (27.9 mill. ha) and other wooded lands (2.3 mill. ha) cover 74% of Sweden’s land area by land-use class according to the Swedish Forestry Act (Nilsson et al. 2025). The country hosts both large areas of lowland ecoregions with a long history of high forest management intensity (Levers et al. 2014), and mountainous areas containing the European Union’s last large intact forest landscapes (Jonsson et al. 2019). The transition to effective wood production was first stimulated by the Swedish iron industry’s needs for charcoal to drive Sweden’s mining industry (Obbarius 1845), followed by an increasing demand for raw material for the forest industry in the late nineteenth century (Lundmark et al. 2013). This led to the process of gradually transforming once naturally dynamic forests (e.g. Pennanen 2002) into an increasingly effective wood production system during the twentieth century (Yrjölä 2002). As a consequence, only few remnants intact forest landscapes remain, and with 97% of forests being managed by even-aged clear-felling systems with rotation forestry (Angelstam et al. 2022).

**Fig. 1:**
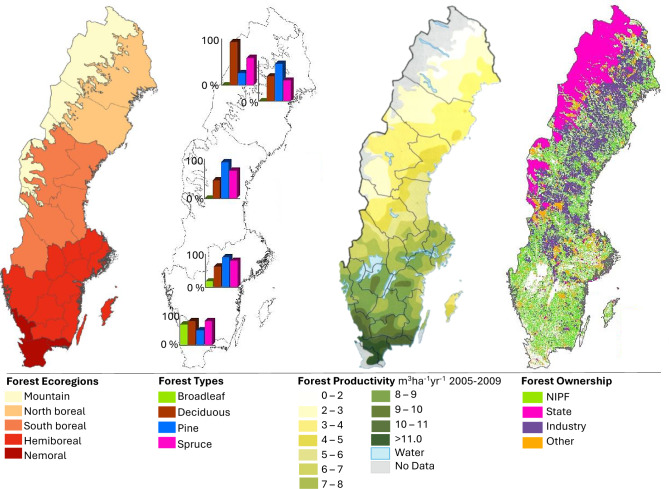
Division of Sweden’s 21 counties into five ecoregions (left), histograms showing the proportions of the four forest types with groups of common tree species in the five ecoregions (centre left), site class forest productivity (centre right), as well as 4 categories of land ownership (NIPF stands for non-industrial private forest (right))

Sweden is divided into five ecoregions ranging from mountain forests in the northwest via lowland north and south boreal and hemiboreal forests, and to nemoral forests in the south, all with characteristic tree species mixtures (Fig. 1). This is linked to forest productivity, which varies > tenfold in Sweden, and is also linked to forest ownership categories (Fig. 1). All but the state are focusing on wood sustained yield production.

### Mapping forest habitats and their quality (Topic 1)

#### Tree species that can be mapped wall-to-wall

In Swedish forest policy (Direktiv 2024), conservation of viable populations of naturally occurring forest species represents a vision of naturalness. This requires addressing representative forest land-cover types and their respective tree species composition. These can be categorised by different principles like associations to local site conditions and disturbance regimes, and with different thematic resolutions. Analysing the range of natural forest disturbance regimes in northern Europe Angelstam (1998), Berglund and Kuuluvainen (2021), Kuuluvainen et al. (2021) and Manton et al. (2025) divided these disturbance regimes into three groups, namely cohort dynamic, succession, and gap dynamic.

The national land-cover database in Sweden (Naturvårdsverket 2019, 2020) maps a total of seven tree species and species admixtures. However, to address the variable thematic precision of the mapping of forest types (Nilsson et al. 2020), we merged some individual and groups of tree species (see Table 1). This resulted in four representative coarse tree species land covers with different natural dynamics: (1) pine (*Pinus sylvestris*) representing cohort dynamic; (2) spruce (*Picea abies*) and mixed-conifer stands, as well as (3) mixed conifer-deciduous and deciduous trees like birches (*Betula* spp.) and aspen (*Populus tremula)*, both groups representing succession; and (4) broadleaf shade-tolerant species like beech (*Fagus sylvatica),* elm *(Ulmus glabra),* linden *(Tilia cordata*), representing gap dynamic. This diversity is in stark contrast to the monolithic even-aged rotation forestry approach that focuses on the two conifer species (Svensson et al. 2022).

**Table 1:** Forest type based on coarse tree species groups defined by the EU habitats directory (in brackets codes according to European Commission (2023)) and NMD (National Land Cover Data) forest types (Naturvårdsverket 2019, 2020). These were merged into four groups based on validation of the NMD data with field data from the National Forest Inventory following the recommendations of (Nilsson et al. 2020)

Forest type	Land-cover type according to the EU habitat directive	NMD Code
Pine	Pine (9010)	111, 121
Spruce	Spruce (9010) Mixed conifer (9010)	112, 113 122, 123
Deciduous	Mixed conifer-deciduous (9010)	114, 124
	Trivial deciduous (9010, 9030, 9040, 9080, 91E0)	115, 125
Broadleafs	Broadleafs (9130, 9110, 9160, 9190) Deciduous with broadleafs	116, 117 126, 127

#### Creating relative naturalness rank maps with 1-ha resolution

Modelling of the relative forest naturalness rank from 0 to 1 for 1-ha pixels in Sweden was made by Bubnicki et al. (2024). This wall-to-wall map is valid for 2019 and covers 21.9 million ha, excluding areas that are classified as temporary non-forested in the national land cover database termed “NMD 2018” (Naturvårdsverket 2019, 2020) and pixels with less than 50% forest cover. The available training data in terms of areas with HCVF characteristics documented in the field represent the situation in 2018 (Bovin et al. 2017) and updated in 2019.

Throughout this article, we thus deal with two categories of forests with high conservation relevance. The first has been subject to field surveys of forests to determine their high naturalness, both protected and not protected. The short name for this is HCVF. The second are those defined by a model that predicts wall-to-wall different levels of forest naturalness in Sweden. The short name for this is naturalness rank from 0 to 1.

Focusing on the end of year 2023, we updated this database by removing the clear-cuts mapped annually by the Swedish Forest Agency, and other kinds of past and recent forest canopy loss documented in the Global Forest Change (GFC) database about forest loss and gain (Hansen et al. 2013). This involved clipping the naturalness rank database with the union of all fellings and canopy losses updated. We thus removed, in addition to temporary non-forest areas (5.2 mill. ha), the union of GFC loss and gain for 2000–2023, and clear-cut areas for 2000–2023 (in total 5.1 mill ha), located outside temporary non-forested areas in the “NMD 2018” database, thus leaving 16.8 mill ha in the naturalness rank database. The total area of forest in Sweden according to the national land-cover data was 27.1 million ha.

The validation of the Bubnicki et al.’s (2024) model using external and independent spatial datasets (i.e. those that have not been used for model training) showed that relative average probability values of 0.7 (CI95 high 0.61–0.78) and higher matched forest stands characterised as HCVF by the National Forest Inventory and in state company Sveaskog’s forest management plans, while values below 0.3 (CI95 low 0.28–0.35) and lower matched stands characterised as focusing on high sustained yield wood production. Values from 0.3 to 0.7 represented intermediate naturalness ranks.

Overall, in Sweden the mean naturalness ranks for the different types of formal protection (SCB 2025) on forest land were 0.76 for national parks, 0.65 for nature reserves, and 0.51 for both biotope protection and conservation agreements. Voluntary set-asides are known to have lower naturalness rank compared to formal protection (Kyashenko et al. 2022) and had a mean naturalness rank of 0.40 (Jonas Dahlberg, pers. comm.). For the subsequent analyses supporting spatial prioritisation of area protection and nature restoration, we created two classes of relative probability of forest naturalness, viz. lower at 0.5–0.69 and higher at ≥  = 0.7, respectively.

### Habitat suitability index modelling (Topic 2)

Habitat suitability index modelling can be described as a filtering approach to assess whether particular focal species’ requirements are met or not in a landscape (e.g. Manton et al. 2005). Another approach is to define a set of virtual species that capture the habitat demands of a selected group of species (Mikusiński and Edenius 2006). Securing sufficient areas of functional habitat networks for representative land-cover types of different forest types with sufficient quality requires sufficient patch sizes for a home range of focal species (e.g. Angelstam et al. 2004). Additionally, the networks of patches should be functionally connected, i.e. that species with different dispersal abilities can navigate between suitable habitat patches across a given area, in contrast to structural connectivity meaning the physical arrangement of habitat patches. Patches should thus not be too sparsely distributed throughout the landscape (Hanski 2015; Pither et al. 2023). This suite of factors affecting habitat network functionality is consistent with Lawton’s (2010) recommendation to focus on patch quality, patch size and functional connectivity regarding the ecological system. Additionally, the longevity of a particular habitat needs to be considered (Rosenvald and Lõhmus 2023). For example, the longevity of early more or less short successional stages after fire or windthrow may be the order of months and years for some species (Kuuluvainen and Gauthiers 2018), while late developmental stages may last for hundreds of years.

Due to their strong affinity to forest structures reflecting high levels of naturalness of preferred forest types and high functional habitat network connectivity, we used a suite of forest birds (Angelstam et al. 2004; Mikusiński et al. 2018) as focal species (Lambeck 2002). The selection of focal species included 16 forest species of conservation concern (Skogsstyrelsen 2022). Their habitat requirements in terms of dominating tree species, forest type, habitat patch size requirement and minimum landscape habitat proportion are summarised in Appendix S1. Based on their habitat patch size requirements, they were divided into two groups of virtual species (sensu Mikusiński and Edenius 2006); (1) birds with lower habitat area requirements (> 5 ha) (e.g. the guild of forest tits (*Paridae*)) and (2) birds with higher habitat area requirements (> 100 ha) (e.g. three-toed woodpecker (*Picoides tridactylus)* and Siberian jay (*Perisoreus infaustus*)).

The analytic steps for habitat suitability index models included: (1) identification of all individual habitat patches with different levels of naturalness, (2) selection of sufficiently large patches for presence of a given group of virtual species, and (3) selection of tracts with different functional connectivity of multiple patches at the local landscape scale (Table 2). Results were presented for the five Swedish forest ecoregions used in official statistics on protected nature (SCB 2025).

**Table 2:** Steps in the habitat suitability modelling approach applied in this study. This resulted in a total of 64 outputs

Step 1		Step 2		Step 3
All forest habitats		Sufficiently large patches		Patch concentration
1a: Forest type (pine, spruce, deciduous, broadleafs)	1b: Naturalness rank (> 0.5 and > 0.7))	2a: Inter-patch distance requirements (200 m and 1000 m)	2b: Area requirements (5 ha and 100 ha)	3: Fragmentation level (10%, 30%) in 1 and 25 km^2^-landscape windows
4 models	2 models	2 models	2 models	2 models

First, we created GIS (geographical information system) themes for the four representative forest types (pine, spruce, deciduous, broadleaf; see Table 1) mapped with acceptable thematic quality (Nilsson et al. 2020), each divided into two naturalness rank categories with relative probabilities of 0.5–1.0 and 0.7–1.0, respectively (Bubnicki et al. 2024). Second, to identify suitable habitat patches, because individuals of different bird species can move to varying extents in the matrix between adjacent habitat patches, we grouped all the habitat patches for each given forest land-cover type (Table 1) separately. As acceptable inter-patch distances for resident boreal forest birds’ movement across adjacent patches to satisfy their resource needs (e.g. Jansson and Angelstam 1999, Åberg et al. 2003), we chose 200 m (i.e. 100-m buffer) for the less area-demanding species and 1000 m (i.e. 500-m buffer) for the more area-demanding species. This was derived by (1) selecting all patches of a land-cover theme, (2) adding the respective buffers to the patches to create clusters of each focal land-cover theme and buffer, and (3) removing the non-habitat buffer from the patch cluster. We then selected aggregated clusters with habitat patches that meet minimum habitat size requirements of the less or more area-demanding focal species. The patch sizes chosen were 5 ha and 100 ha, respectively. This patch size selection matches the mean demands of each of the less and more area-demanding species groups (Angelstam et al. 2004, 2020; Appendix S1). Third, as a simple measurement of functional connectivity, we used the metric of suitable habitat density (Auffret et al. 2015). This calculates the area percentage of habitat patches over a featured landscape filtered by a moving window. We used nearest neighbourhood analysis (the ArcGIS term focal statistics) to assess the landscape-level functional connectivity of the patches using moving window sizes of 1 km^2^ corresponding to the species with smaller area requirement (5 ha) and 25 km^2^ for those with larger area requirements (100 ha), respectively. Finally, following the European Commission’s protection targets and empirical evidence (e.g. Hanski 2011), we applied widely used minimum habitat availability thresholds of 10% and 30% to define suitable functional habitat networks at the landscape level (Table S2) for each of the two groups of virtual species and four groups of tree species (see Manton et al. 2005; Angelstam et al. 2020). This stepwise filtering approach resulted in 64 outputs presented as maps representing different focal species requirements (Table 2).

### Formal protection of forests with high naturalness ranks (Topic 3)

SCB (2025) reports on four groups of set-aside areas: formally protected, voluntarily set-aside, unproductive forests and nature considerations. Of these only formally protected areas satisfy IUCN’s criteria for area protection (Dudley 2008). The proportion of different IUCN-protected area categories of Sweden is 1.5% for category Ia (strict reserve), 7.0% for category Ib (wilderness area), 1.5% for category II (national park), and a total of 1.1% for categories III, IV and V (natural monument, habitat/species management area, protected landscape); category VI (with sustainable use of natural resources) is not applied (Naturvårdsverket 2021). We analysed the extent to which forests with different levels of predicted relative naturalness (Bubnicki et al. 2024) are formally protected. We used the updated GIS layer available on request from the Swedish Environmental Protection Agency valid for the end of 2023. It includes ten categories of formally protected areas on forestland (SCB 2025), which are grouped into three non-overlapping categories, viz. formally protected (national parks and nature reserves), other kinds of formal protection (e.g. biotope protection, two types of conservation agreement, Natura 2000, Swedish Fortifications Agency) and in the process of being formally protected (three types with formal decision taken, but the administrative process is ongoing). This GIS layer for formally protected areas was combined with the ranking of forest naturalness in 2019 (Bubnicki et al. 2024), which was updated by removing clear-cut areas to match the end of 2023. We present how much of two intervals of relative naturalness (0.5–0.69 and 0.70–1.0) is formally protected vs. not protected among the five Swedish forest ecoregions (SCB 2025).

### Identification of areas for potential protected area designation (Topic 4)

To address the opportunity for Sweden to meet policy targets for area protection by additional set-asides in different ecoregions, one option is to estimate the amount of forest with high naturalness ranks (i.e. Bubnicki et al. 2024), both inside and outside the field-inventoried forests with high levels of naturalness defined by continuous forest mapping (T. Ek, pers. comm.). To identify areas for potential protected areas designation, we identified areas with the highest potential in terms of naturalness by overlaying the GIS layer with high naturalness ranks and the GIS layers with unprotected HCVFs surveyed in the field (i.e. woodland key biotopes (“NBi”)), forests with protection qualities and old-growth forests (“SNUS”), core areas for area protection identified by the county administrations (“VK”) and natural mountain forest.

### Amount, location and ownership of hotspot areas (Topic 5)

To compare the opportunities for different approaches to conservation planning, contiguous forest patches with naturalness ranks 0.5–1.0 were divided into three patch sizes, viz. 5, 500 and 50 000 ha. These patch sizes reflect two complementary dimensions of biodiversity conservation: (1) different area requirements of focal species individuals, species assemblages and forest disturbance regimes, and (2) different levels of ambition ranging from habitat patch occupancy via viable populations to ecosystem resilience (e.g. Diaz et al. 2020; Angelstam et al. 2023). Patches exceeding 5 ha match the minimum requirement for the presence of individuals of the least demanding focal species (Appendix S1). Similarly, assuming a long-term edge effect of 100 m (e.g. Aune et al. 2005) we argue that forest patches should exceed 5 ha in size to retain their core area qualities in the long term (see also Nagel et al. 2025). With limited habitat network functionality due to small and fragmented habitats, as in most of Sweden (Angelstam et al. 2020), an alternative for biodiversity conservation is to focus also on larger contiguous patches of forests with high levels of naturalness. The habitat patch size at which species–area relationships level out provides a coarse proxy of the necessary minimum patch size for species assemblages. A review of estimated minimum requirement of focal boreal and temperate bird species (Angelstam et al. 2004, 434) suggested a mean patch size of 150 ha and a mean probability of 0.30 of habitat patch occupancy of focal bird species in a particular forest landscape. This suggests a necessary minimum mean patch size for a complete local bird assemblage of ca. 150/0.3 ~ 500 ha. The 50 000-ha patch size is motivated in three ways. The first is focused on the total size of an individual functionally connected habitat network cluster within the area corresponding to that required to host an effective population size of 100 individuals (Meffe and Carroll 1994). The review by Angelstam et al. (2004, 434) about parameter values for habitat suitability index modelling showed that the median estimate for 15 focal bird species was 50 000 ha/500 km^2^ for an effective population. The second is the study by Hintsanen (2024) on the effects of patch size of naturally dynamic forest remnants on the long-term population viability of resident birds in Finland, which suggested a minimum landscape size requirement of 450 km^2^. Third, 50 000 ha that matches the concept of large intact forests (Potapov et al. 2017; Jonsson et al. 2019) can be interpreted as a minimum dynamic area allowing natural disturbance regimes and ecological resilience.

The spatial distribution and amount of these three patch sizes are presented for the five Swedish ecoregions and four different forest ownership categories. This was made by using ArcGIS to overlay the main forest owner categories (non-industrial private, industry, other private and state) with different naturalness ranks. Finally, for each ecoregion we used GIS to estimate the proportions that were formally protected at the end of 2023, divided into two groups of naturalness rank ranges 0.5–0.69 and ≥ 0.7, respectively.

## Results

### Mapping forest habitats and their quality (Topic 1)

**Fig. 2:**
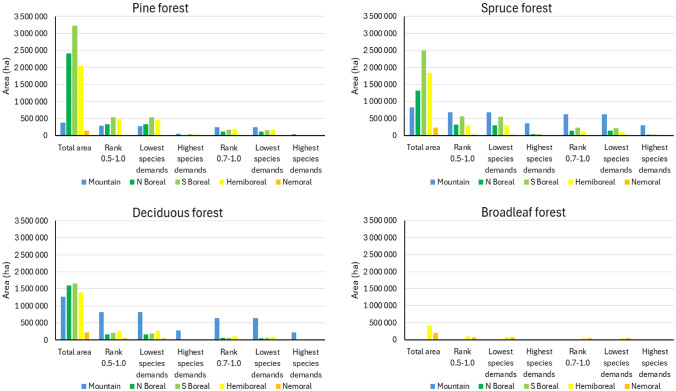
Graphs illustrating the area extent of four different forest types, and the constituent areas of two different naturalness ranks according to Bubnicki et al. (2024), as well as the area satisfying lower and higher patch size and functional connectivity demands of focal species. Numbers behind graphs are presented in Appendix S2, Table S3

**Fig. 3:**
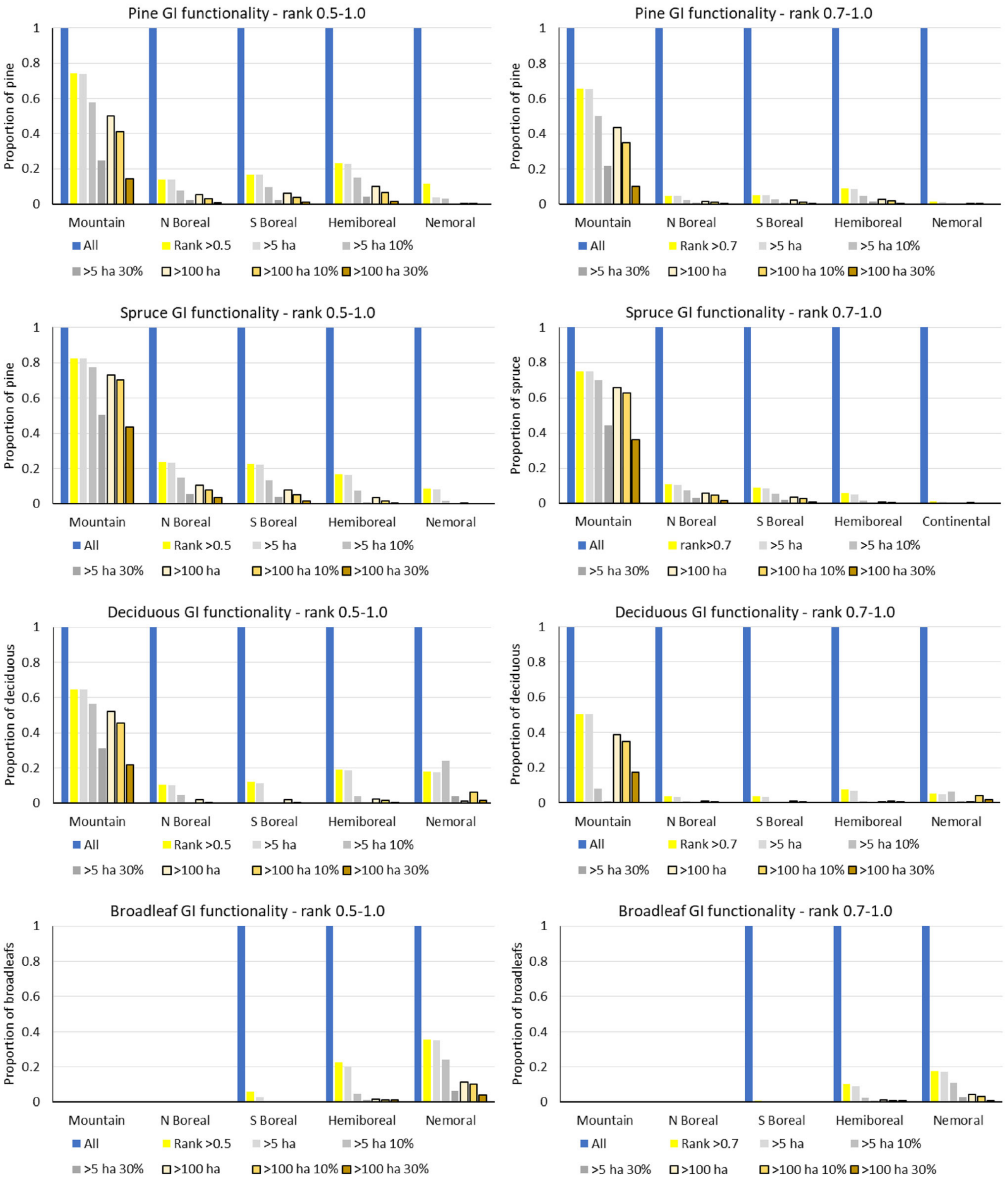
From top to bottom, the proportions of all pine, spruce, deciduous and broadleaf forest with naturalness ranks being ≥ 0.5 (left) or ≥ 0.7 (right) satisfying increasing area requirements (5 to 100 ha) with increasing functional connectivity as green infrastructure (GI) in local landscapes (10 to 30%) in the five Swedish forest ecoregions, respectively; raw data behind proportions in Table 3

Overall, the total area of pine, spruce and deciduous forest was highest in the south boreal ecoregion, whereas broadleaf forests were confined to the hemiboreal and nemoral ecoregions (Fig. 2, Table 3). There were, however, large differences in the summed proportions of different forest naturalness ranks among the five ecoregions (Fig. 3). The average proportion of all four forest types in all of Sweden was 0.20 to 0.28 for natural rank 0.5–1.0 and 0.09 to 0.26 for the rank interval 0.7–1.0 (Table 3). In the mountain ecoregion the range of relative naturalness ranks 0.5–1.0 and 0.7–1.0 made up 74 to 82% and 75 to 89%, respectively. For the other ecoregions below the mountains the total proportions ranged from 14 to 26% for the wider range of relative naturalness ranks (0.5–1.0), but only from 5 to 12% of the narrower (0.7–1.0) range (Table 3).

**Table 3:** Total area, and area of four forest types with different naturalness rank ranges in the five Swedish forest ecoregions

Ecoregion	Pine			Spruce			Deciduous			Broadleaf		
	Total area	Rank 0.5–1.0	Rank 0.7–1.0	Total area	Rank 0.5–1.0	Rank 0.7–1.0	Total area	Rank 0.5–1.0	Rank 0.7–1.0	Total area	Rank 0.5–1.0	Rank 0.7–1.0
Mountain	379 423	281 439	249 305	816 084	672 318	613 672	1 273 679	822 603	641 187	0	0	0
N Boreal	2 404 395	337 699	113 475	1 321 810	313 258	143 476	1 597 965	166 738	56 105	0	0	0
S Boreal	3 221 981	538 534	167 074	2 488 757	557 872	224 240	1 658 730	201 506	63 738	16 652	999	123
Hemiboreal	2 047 708	473 741	179 752	1 834 537	30 4715	104 018	1 395 994	267 136	103 030	409 638	92 632	42 321
Nemoral	138 498	16 163	2049	222 990	19 108	2671	223 229	39 917	11 889	191 937	68 120	33 606
SWEDEN	8 192 005	1 647 576	711 655	6 684 178	1 867 271	1 088 077	6 149 597	1 497 900	875 949	618 227	161 751	76 050
Proportion in Sweden		0.20	0.09		0.28	0.16		0.24	0.14		0.26	0.12
Proportion in mountain		0.74	0.89		0.82	0.75		0.65	0.78		NA	NA
Proportion below mountain		0.17	0.06		0.20	0.08		0.14	0.05		0.26	0.12

### Habitat suitability index modelling (Topic 2)

Of all the modelling variants, we focus on results covering lower-to-higher demands of virtual focal species in terms of the relative naturalness rank, habitat patch size and difficulties to cope with spatially fragmented habitats. The habitat modelling clearly shows that the relative functionality of pine, spruce and deciduous forest networks were much higher in the mountain ecoregion compared to the lowland ecoregions (Fig. 3). In the other ecoregions, pine performed best in the south boreal ecoregion, while the performance of spruce habitat declined from north to south. Outside the mountain ecoregion, the deciduous forest networks improved from the north boreal to the nemoral ecoregions. Finally, for broadleafs, which are confined to the hemiboreal and nemoral ecoregions, the habitat network functionality increased towards the south.

**Fig. 4:**
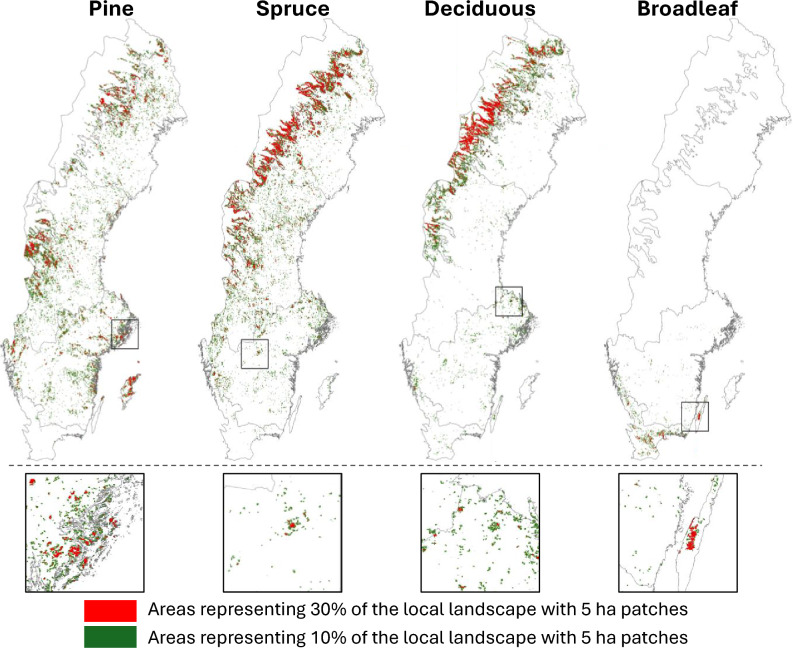
Maps of pine, spruce, deciduous and broadleaf forest (top) showing the location of functional habitat networks for virtual species with patch size requirement of 5 ha, and requiring patch densities of 10 and 30% in local neighbourhoods (1 km^2^ or 25 km^2^), respectively (see Table 2). Inset maps (bottom, 100 by 100 km) show examples of regional hotspots at a spatial extent suitable for landscape planning

In Fig. 3, results from the three modelling steps for focal species with lower and higher conservation ambitions, respectively, are presented as proportions for the four forest types and two levels of naturalness. For the least demanding species, the mean proportions of all pine, spruce and deciduous tree species with natural ranks 0.5–1.0 below the mountain forest that satisfy all modelling steps was on average 9, 9 and 8% (Fig. 3, left-hand panels), respectively. In the mountain ecoregion, the proportions were 58, 77 and 57%. For the most demanding species (Fig. 3, right-hand panels), the corresponding proportions were 0, 1, 0, and 10, 36 and 17%. Broadleaf forests were by and large confined to the hemiboreal and nemoral ecoregions. Here the results from habitat suitability index modelling were on average 3% for the least demanding species and 0% for the most demanding species. The results of the different steps in habitat suitability index modelling expressed by ecoregions can also be presented as maps for use by planners (Fig. 4).

### Formal protection of forests with high naturalness ranks (Topic 3)

The proportions of formally protected areas in the five ecoregions were two to four times higher for the higher (0.70–1.0) compared to the lower range (0.50–0.69) of predicted relative naturalness (Table 4). There were considerable differences in the proportion of formal protection among the five ecoregions. The mountain ecoregion had the highest proportion of formal protection (27 and 57% for the two ranges of predicted naturalness), followed by the two boreal ecoregions. In the hemiboreal and nemoral ecoregions, the corresponding proportions ranged from 3 to 16%, respectively.

**Table 4:** Proportions of formally protected areas in forests merged to include all patch sizes and tree species with the relative naturalness ranks 0.50–0.69 and 0.7–1.0 in five ecoregions, respectively. Data about formal forest protection for 2023 from the Swedish Environmental Protection Agency

Ecoregion	Relative naturalness rank range 0.50–0.69		Relative naturalness rank range 0.70–1.0	
	Proportion formally protected	Area extent (ha)	Proportion formally protected	Area extent (ha)
Mountain	0.269	361 368	0.568	1 877 172
N Boreal	0.127	588 821	0.370	352 233
S Boreal	0.050	966 666	0.201	513 233
Hemiboreal	0.034	843 955	0.102	512 302
Nemoral	0.043	117 462	0.155	59 729
SWEDEN	0.088	2 878 272	0.411	3 314 669

### Identification of areas for potential protected area designation (Topic 4)

**Fig. 5:**
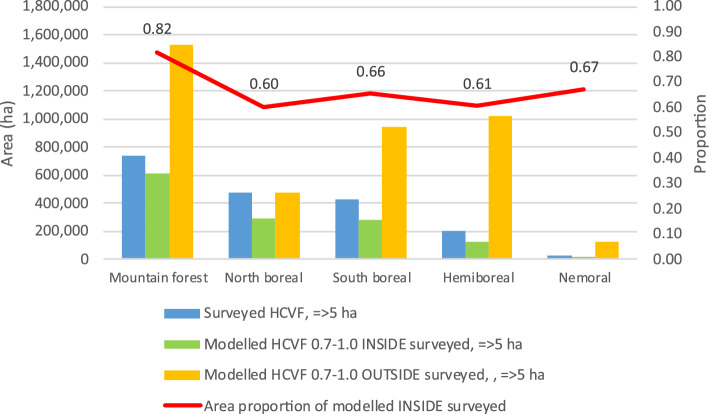
Analysis supporting identification of areas for potential protected area designation. First, the line with numbers shows the proportion of areas with modelled high forest naturalness (Bubnicki et al. 2024) compared to the area of High Conservation Value Areas ≥ 5 ha in size surveyed in the field. Second, the naturalness modelling identified considerable additional areas with high modelled naturalness outside surveyed HCVFs. There is thus a potential for establishing additional protected areas

Comparing the proportion of HCVFs surveyed in the field on the one hand and the proportion of those that were identified by modelling of naturalness with a cut-off value range of 0.50–1.0 on the other, shows that modelling identified on average 70% (range 60–82%) of what was found in the field (Fig. 5). However, modelling also identified considerable areas with naturalness rank 0.5–1.0 located outside HCVF areas identified in the field. For mountain forest, this was 2.5-fold (1524 kha), for north boreal also 2.5-fold (473 kha), for south boreal this was 2.2-fold (942 kha), for hemiboreal this was 4.9-fold (1018 kha), and for nemoral this was 5.1-fold (126 kha).

### Amount, location and ownership of hotspot areas (Topic 5)

**Fig. 6:**
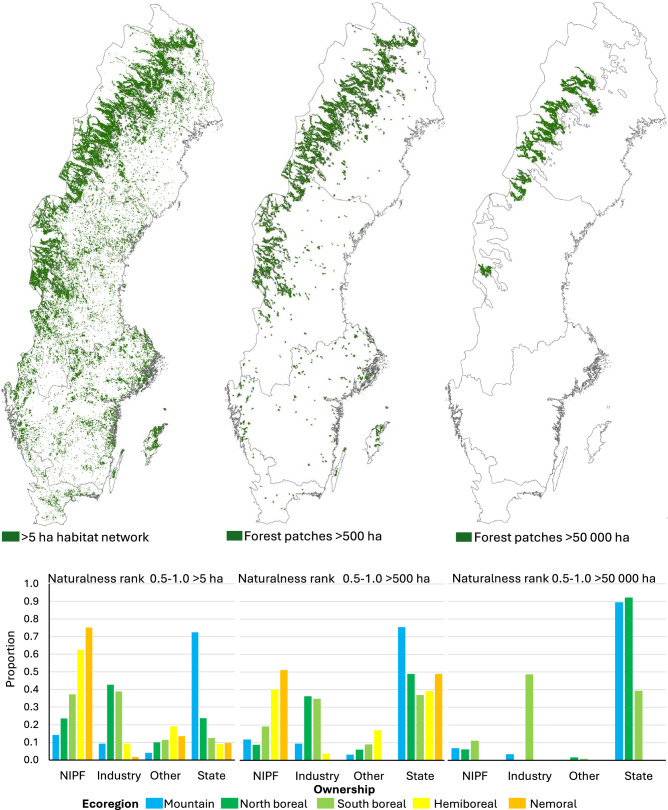
Maps of forests with natural ranks 0.5–1.0 showing tracts for virtual species with minimum patch size requirement of 5 ha, and requiring patch densities of 10% in local neighbourhoods for all tree species in Fig. 4 merged (top left), forest centroids for 780 conservation hotspots with and sized > 500 ha (top centre), and six polygons sized > 50 000 ha (top right) for all forest types. Histograms show proportions of forest owner categories in the five Swedish ecoregions hosting forest patches > 5 ha (bottom left), > 500 ha (bottom centre) and > 50 000 ha (bottom right) for all tree species with naturalness ranks 0.5–1.0 for all tree species merged (data in Tables S4, S5 and S6 in Appendix S2)

As shown in the analyses of functional connectivity, outside the mountain forest ecoregion, only a few per cent of the total area of different forest types form functional habitat networks. This is valid even with a focus on the least demanding focal species used in our habitat suitability modelling approach. To implement current policy targets for formal protection more of forests with high naturalness ranks thus need to be set aside. Focusing on the spatial distribution of forest patches sized > 5 ha, > 500 ha and > 50 000 ha with naturalness ranks 0.5–1.0 as hotspots, the maps in Fig. 6 clearly suggest that the opportunities for securing higher levels of conservation ambitions linked to larger hotspot areas is very limited below the mountain forest region.

We also analysed how the area of different patch sizes was distributed among patch size classes in Sweden’s forests (histograms in Fig. 6). For NIPF (non-industrial private forest) owners and industry, the proportion of forests with a high naturalness rank declined with increasing patch size, from 32 and 20% for 5 ha, 13 and 13% for 500 ha, and to 7 and 7% for 50 000 ha, respectively. State ownership increased from 38% in patches > 5 ha, to 69% in patches > 500 ha, and 86% in patches > 50 000 ha. For all three patch sizes of forest with high levels of naturalness (rank 0.5–1.0), there were large differences among the distributions of different forest ecoregions among forest ownership categories (Fig. 6).

## Discussion

### Environmental history as a key driver of habitat network functionality

Our analyses show that the extent to which habitat networks for conservation of species that require high levels of naturalness are functional in Sweden is clearly related to the environmental history context (sensu Worster 1989). This separates the hinterland mountain ecoregion with unproductive forest (Jonsson et al. 2019) from the rest of Sweden with productive forests in lowlands (Fig. 1). This gradient also reflects different conservation benchmarks linked to natural forest dynamics in the north (e.g. Berglund and Kuuluvainen 2021), and traditional agricultural woodland landscapes in the south (Eriksson 2021), respectively.

The two southernmost Swedish nemoral and hemiboreal ecoregions have the longest history of providing goods, services and values by maintaining multifunctional rural village systems in forest and woodland landscapes for many centuries, which were well maintained until the early twentieth century (e.g. Helmfrid 1961; Gadd 2011). Outfield areas inside the forest mask were managed with low intensity and therefore even still have scattered remnants of stands and trees with high levels of naturalness (e.g. Eliasson and Nilsson 2002). Additionally, unless replaced with planted conifers (Lindbladh et al. 2014), infield areas outside the forest mask may contain wooded grasslands with high conservation value veteran trees (Sverdrup-Thygeson et al. 2017).

Further north, the northern hemiboreal and southern boreal forest ecoregions have been subject to long-term sustained yield forestry in mining districts for more than two centuries (Angelstam et al. 2013). Beginning in the nineteenth century, the southern and northern boreal ecoregions became subject to a gradually expanding timber frontier moving upstream rivers along valleys, and northwards. The aim was to transform naturally dynamic boreal forests to cropping systems to access and to produce wood more effectively (Törnlund and Östlund 2006; Angelstam and Manton 2021). The remote mountain forest ecoregion is currently located at the edge of this frontier. Consequently, this ecoregion is characterised by having high naturalness with large patch sizes and good functional connectivity at the landscape level (Angelstam et al. 2020; Svensson et al. 2022). The higher levels of naturalness in the mountain forest ecoregion ensures functional habitat networks for otherwise rare species (see Angelstam et al. 2020; Hintsanen 2024), making it critically important for conservation not only in Sweden, but also at the international level by forming most of the last few large intact forest massifs in the EU (e.g. Jonsson et al. 2019).

Our analyses show that Swedish conservation efforts have succeeded to formally protect the highest proportions of forests with high levels of naturalness, mostly in the mountain ecoregion. However, in the forest regions used for wood production below the mountain ecoregion, which cover most of the forest mask in Sweden, the area proportion of formally protected areas is very low (i.e. 3 to 5%; see SCB 2025) compared to agreed international and EU policy targets (i.e. 30%; see CBD (2022) and European Commission (2020)). Additionally, compared to the mountain ecoregion, in the four lower-elevation ecoregions, our analyses show that the proportion of functionally connected habitat clusters is very low, especially for more demanding species. Thus, only 8–9% of pine, spruce and deciduous forest with high naturalness ranks, respectively, satisfy the requirements of the least demanding species, as opposed to 57–77% in the mountain forest ecoregion. For nemoral forests, the proportions of functional habitat works were < 3%. For the most demanding focal species, the proportions were even lower. Functional connectivity therefore urgently needs to be improved by strategic set-asides of the remaining unprotected forests with high levels of naturalness, especially because the matrix around remnants of those are unfavourable for conservation of specialised species (e.g. Kouki and Väänänen 2000). It is therefore crucial to assess the net effect of conservation measures and spatially expanding forest management intensity. The current negative net effect of these factors (e.g. Angelstam and Manton 2021) is currently aggravated due to the combined effects of cutbacks in funding to establish new protected areas (Proposition 2021/22:58), increasing rate of fellings relative to growth and declining age of final felling (Nilsson et al. 2025).

### Assessing habitat network functionality

#### Forest habitats and their quality

There is a mismatch between the richness of forest types that can be identified, and those that can be mapped wall-to-wall with high spatial resolution. Forest ecosystems vary ecoregionally in terms of the distribution and abundance of different tree species and can be categorised in several ways. According to rules for reporting to the EU the state of forest habitats (Naturvårdsverket 2011) Sweden hosts 15 different forest types and one type of cultural woodland. Based on natural disturbance regimes, topography and soils, and traditional management of cultural wooded grasslands, Angelstam and Andersson (2001) recognised 12 different forest types and two cultural woodland types. However, not all forest types defined by tree species can be mapped wall-to-wall matching the spatial extent of forest stands with a few ha in size. Validation of land-cover types derived from remote sensing with data from the Swedish National Forest Inventory show that only four broad forest types can be mapped wall-to-wall in Sweden with acceptable thematic precision (Nilsson et al. 2020).

Fortunately, the relative likelihood of forest naturalness can be mapped (Bubnicki et al. 2024). We used the first available database mapping contiguously wall-to-wall the relative probability of naturalness from low to high with a spatial resolution of 1 ha in Sweden. A major strength of the approach lies in mapping continuous probabilities of the forest areas being assessed, enabling nuanced analyses across gradients in forest management intensity. Being validated with independent data from the Swedish National Forest Inventory, and field-based forest data from the state forest company Sveaskog’s stand register (Bubnicki et al. 2024), it is of high thematic quality. Validation of spatial modelling using bird monitoring data show good match between predictions and bird species of conservation concern (e.g. Angelstam et al. 2020; Bakx et al. 2023; Kost and Olsson 2025; Yildirim 2026).

#### Integrating patch size and functional connectivity using habitat suitability index modelling

We used habitat suitability modelling based on knowledge about birds’ habitat requirements to assess habitat network functionality. Birds are well studied, are charismatic and effective to communicate evidence-based ecological knowledge about them, and about the results from the different steps in habitat modelling procedures. Therefore, different types of habitat suitability modelling using birds have been applied (e.g. Manton et al. 2005; Appendix S1). However, outcomes of modelling are sensitive to the selection of variables and parameter values representing different focal species (Manton et al. 2005). Rather than pointing out the habitat requirements of a particular bird species, we therefore used a virtual species approach (e.g. Mikusiński and Edenius 2006) and reviewed qualitative and quantitative requirement of two suites of bird species reflecting lower and higher demands concerning habitat quality, patch size and ability to cope with fragmentation (for a detailed discussion, see Appendix S1).

#### Longevity of dynamic habitat patches

Forest ecosystems are dynamic (Kuuluvainen et al. 2021), in both time and space. While forests with gap and cohort dynamics are characterised by continuous tree cover of older trees, forests with stand-replacing disturbances go through different successional stages, often with remnant structural legacies (Manton et al. 2025). This begins with short-lived habitat like burned wood and dying trees, followed by a phase with shade-intolerant deciduous trees, and eventually a long phase with more stable conditions and shade-tolerant tree species (Kuuluvainen and Gauthier 2018). For species with good ability for habitat tracking, this implies that it is necessary to consider the total area extent of successive multiple minimum dynamic areas. Designing such dynamic reserves with shorter or longer durations means that the area requirement for persistence of species in viable populations is higher in the long term than in the short term (Bengtsson et al. 2003).

### Conservation ambitions determine assessments of habitat functionality

In this study, we made habitat suitability index models using both lower and higher demands in terms of bird species’ habitat quality, habitat patch size and ability to cope with habitat fragmentation of particular forest types. The results were presented as multiple outputs representing different steps of the filtering in the modelling process. The habitat requirements of the two groups of virtual species had a considerable effect on assessments of what proportion of a particular forest type is forming a functional habitat network with high naturalness ranks. This matches results from long-term monitoring of bird species, which shows that forest specialists dependent on high forest naturalness are declining while generalists are stable (Bakx et al. 2023). Similar patterns are found also for other taxa (Nordén et al. 2013; Petersson et al. 2023).

Habitat suitability index modelling shows that not only environmental history, but also conservation ambitions of different forest actors and owner categories, affect assessments of whether or not forest habitat patches form functional networks in Sweden. A first level of ambition is habitat patch occupancy, which is illustrated by presence of individuals of species in remnant patches of once widespread naturally dynamic habitats. This level is captured by voluntary certification standards that focus on presence of individuals of species (e.g. Matias et al. 2024), and which industrial and non-industrial forest owners in Sweden abide to. A second level of ambition is that naturally occurring species form viable populations, which should be maintained in the long term. This level is what is prescribed in Swedish forest and conservation policy. Additional levels of ambition are to ensure ecosystem resilience, integrity and health, allowing interactions among species and processes (e.g. Bengtsson et al. 2003), and to ensure ecological resilience (Gunderson 2000; Mina et al. 2022) under scenarios of climate and global economic change. These three different levels of ambition apply both to visions of naturally dynamic forests and cultural landscapes.

These levels of ambition can also be compared to the stepwise filtering approach in habitat suitability index modelling (Table 2). The lowest level of ambition (occupancy) is reflected in the area proportions of different forest types with different degrees of naturalness. The second level of ambition (viable populations) is captured by focusing not only on suitable habitat quality, but also on the area requirements. One can thus focus on species which have smaller area requirements and are less sensitive to habitat fragmentation, or focus on species which have larger area requirements and are more sensitive to habitat fragmentation. The third level of ambition (resilience) focuses on the need for emulating natural disturbance processes (Aszalos et al. 2022; Manton et al. 2025) as a means of securing both renewal of short-lived habitat like deciduous succession after stand-replacing disturbance (Bengtsson et al. 2003) and continuity forests (Kuuluvainen et al. 2021). This can enhance engineering resilience, ecological resilience as well as social–ecological resilience (sensu Nikinma et al. 2020). The latter has implications for modification of traditional forest management models with the aim to enhance understanding of and management for resilient forests (Albrich et al. 2020). In the context of global change, it is urgent to adopt a holistic social–ecological resilience concept (IPBES 2024).

Analyses of habitat suitability in ecoregions with different environmental histories are a powerful way of assessing, both qualitatively and quantitatively, the extent to which reality matches particular levels of conservation ambition or not. This is a foundation for evidence-based learning (Angelstam and Dawson 2025). In contrast, there can be a focus in politics to choose levels of conservation ambition that match narratives desired by powerful actors who focus on intensive wood production, and to ignore the extent to which habitat networks are functional or not. Another kind of abuse is to report data about protected areas for entire Sweden, including the well-protected mountain forest ecoregion, without highlighting that this exaggerates the conservation status of all the other forest landscapes. To spot this kind of cherry-picking of data requires attentive knowledgeable fact-checking communicators and citizens (e.g. Reed et al. 2025).

### The need for landscape planning

The poor functionality of habitat networks for the maintenance of viable populations of natural occurring species calls for increasing the area extent of protected areas, conservation management and nature restoration. The habitat suitability index modelling approach and the resulting thematic maps produced in this study can guide spatial planning for prioritisation of where actions would be most effective.

Regarding protected areas, this study demonstrates that past conservation planning in Sweden has indeed identified and protected forests in areas with higher naturalness than found in the forest landscape in general. Thus, the proportions of formally protected areas in the five ecoregions were two to four times higher for the naturalness rank 0.7–1.0 compared to the lower range (0.5–0.7) of relative naturalness (Table 4). However, on average 59% of forests with naturalness rank range 0.7–1.0 are not formally protected in Sweden, and there are considerable areas of forests with high levels of naturalness in comparison with what has been recorded in field surveys (Fig. 5). Modelling of naturalness ranks is thus an important complement to field surveys. To sustain habitat qualities in protected areas over time, conservation management and nature restoration may be needed. This applies, for example, to early successional stages that require prescribed fire to be maintained (Berglund and Kuuluvainen 2021), and the maintenance of veteran trees in the hemiboreal and nemoral forest ecoregions (e.g. Sverdrup-Thygeson et al. 2017).

We show that, except in the mountain forest ecoregion, habitat patches with high naturalness ranks in Sweden are small and highly fragmented. Thus, satisfying the requirements of viable populations of species by securing sufficient functionally connected habitat area (the second level of ambition) is not likely beyond the immediate local scale for presence of species with very small area requirements (the first level). Securing sufficient forest patch sizes allowing natural disturbance regimes (the third level) is not possible outside the mountain region due to the high level of fragmentation of forest habitats. This suggests avoiding a focus only on conservation of small patches located in tracts with low functional connectivity, and instead to conserve sufficiently large patches able to stand alone as large individual regional hotspots for biodiversity conservation. For example, Sweden has 780 patches with high naturalness ranks and exceeding 500 ha, irrespective of the forest types involved. Concentrating area protection, conservation management and nature restoration to such hotspots is an avenue for securing representation of different forest ecoregions. With a European perspective, intact forest landscapes > 50 000 ha in size (Potapov et al. 2017; Jonsson et al. 2019) in Sweden are by and large already formally protected, and the state is a dominating forest owner of both hotspots areas > 500 ha and intact landscapes > 50 000 ha in size. Indeed, comparing the proportions of state owned (i.e. generally formally protected) forest areas with high naturalness ranks across ecoregions showed that the proportions of patches sized > 5 ha, > 500 ha, and > 50 000 ha amounts to 25, 50 and 74%, respectively. The main conservation challenges are to compensate landowners for securing the last remnants of forests with high levels of naturalness (Fig. 6), and to plan spatially for functional habitat networks in a way that respects different forest ownership categories (see examples in Angelstam et al. 2020).

The functionality of habitat networks is also affected by the level of forest naturalness of the surrounding matrix (Häkkilä et al. 2017; Svensson et al. 2019, 2022). In Sweden, forest harvesting rates relative to growth have increased over recent years (Nilsson et al. 2025). This implies an arms race between the functionality of conservation set-asides and intensification of forestry, unfortunately leading to a net loss of forests with high conservation values (Angelstam and Manton 2021; Määttänen et al. 2022; Räty et al. 2023). Comparing the available growth of wood biomass with harvesting rates during the recent 5-year period shows that while in northern Sweden harvest rates are on average 75% of the available growth, in the southern half of Sweden harvesting rates were on average 112% of the available growth (P.-E. Wikberg pers. comm.). Pilot studies that carry out attempts to restore nature are appearing (e.g. Edman et al. 2024; Wang et al. 2025), being motivated by hopes of future gains in conservation value. However, these are fraught with uncertainties due limited spatial extents, high costs to scale up, and long time to deliver expected outcomes on the ground. To avoid irreversible losses of primary and old-growth forests remnants, it is thus urgent to conserve “the last of the last” forests with high naturalness ranks and suitable locations that still remain (Svensson et al. 2022), as well as to encourage closer-to-nature forest management (Larsen et al. 2022; European Commission 2023) in both the surrounding managed matrix and if necessary in set-aside and protected areas.

With its Linnean heritage pioneering the conservation of biodiversity by naming species, and stimulating comprehensive surveys of habitats for plants, fungi and animals, there is a current puzzling stark contrast in Sweden between solid evidence-based knowledge about the unfavourable state of biodiversity (Skogsstyrelsen 2022) and limited habitat network functionality reported in this study, and the current anti-conservation politics in Sweden (e.g. Chapron et al. 2023). For example, rather than focusing on all aspects of sustainable forest management, the focus of national politics has transitioned to “*increased forest growth and a long-term increased access to sustainable forest biomass*” (Direktiv 2024), and severely reducing efforts supporting biodiversity conservation and climate adaptation (see SOU 2025). Moreover, until now Sweden has chosen to report the favourable reference value for most forest habitats as 20 per cent of a historical reference-based baseline extent (DG Environment 2023). In contrast to the Environmental Objectives Committee’s report (SOU 2025), according to the regulation letter on forest habitats (Naturvårdsverket 2025), the reference value should now be when Sweden joined the European Union, i.e. in 1995. The purpose of this change is to contribute to improved Swedish competitiveness for forestry through reduced regulatory burden and increased comparability with other EU Member States. This implies a paradigm shift from a scientific evidence-based conservation perspective to a political one (e.g. Svancara et al. 2005) driven by forest industry as a strong actor. As unfavourable drivers for functional habitat networks, Dawson et al. (2025) observed major constraints to biodiversity friendly forestry methods in terms of conservative traditions and inadequate advisory services by wood procurers. Suggested remedies were hard and soft policy and economic instruments to incentivise a shift away from a narrow focus on intensive forest management.

## Conclusions

This study presents a comprehensive analysis of relative forest naturalness, habitat suitability and conservation needs across Sweden’s different forest ecoregions. It highlights the critical importance of the mountain ecoregion as a stronghold for biodiversity conservation with high levels of ambition at the European level. We also identify significant challenges for the rest of Sweden with its severely modified and fragmented forests. To safeguard forest biodiversity in Sweden, immediate action is required to protect the remaining large forest patches, enhance their habitat connectivity and implement targeted conservation strategies for conservation management and nature restoration that reflect the unique social–ecological contexts of different forest ecoregions. The future of Sweden’s forest biodiversity depends on concerted collaborative efforts, and appropriate policy instruments including funding for both set-asides and conservation management, that satisfy conservation ambitions within the practical realities of forest landscape management. Honouring the Linnean heritage of biological literacy is an appropriate guiding star.

## Supplementary Information

Below is the link to the electronic supplementary material.

Supplementary file1 (PDF 506 KB)
