# Supplementary material for: Environmental history determines forest habitat network functionality: The need for landscape planning in Sweden
Source: Ambio. 2026 Mar 1;55(8):1845–65. doi: 10.1007/s13280-026-02353-7 (PMC13319304; doi:10.1007/s13280-026-02353-7)
Supplement: Supplementary file 1 — Supplementary file1 (PDF 506 KB) [file 13280_2026_2353_MOESM1_ESM.pdf]

Ambio

Supplementary Information
This supplementary information has not been peer-reviewed

Title: **Environmental history determines forest habitat network functionality – the need for landscape planning in Sweden**

Appendix S1. Review of parameter values for virtual bird species

To assess forest habitat network functionality across Sweden's five ecoregions we used habitat suitability index modelling to integrate species' habitat requirements in different steps (e.g., Manton et al. 2005; Öhman et al. 2011; Orlikowska et al. 2020; Mikusiński et al. 2021) in a spatially explicit manner across five ecoregions (Angelstam et al. 2020). We focused on birds, which are well studied, charismatic and therefore effective to communicate evidence-based ecological knowledge about biodiversity status (Roberge and Angelstam 2006; Roberge et al. 2008; Lõhmus et al. 2017; Fraixedas et al. 2020). Moreover, birds' requirements illustrate well the logic behind the different steps in the habitat modelling procedure (Gregory et al. 2005; Virkkala et al. 2022).

However, outcomes of habitat suitability index modelling are sensitive to the selection of parameter values representing different focal species' requirements (Angelstam et al. 2004; Manton et al. 2005). Rather than pointing out the habitat requirements of particular bird species, we therefore used a virtual species approach (e.g., Mikusiński and Edenius 2006). This approach integrates key spatial habitat requirements, namely lower and higher demands concerning patch size for a home range, and species' ability to cope with fragmentation for 16 bird species listed by the Swedish Forest Agency as potentially affected by forestry (Table S1). These bird species represent forests with high levels of naturalness dominated by four different tree species (or groups thereof), and having lower to higher habitat patch size demands that range from 5 to 300 ha (Table S1).

Based on this variation in patch size requirements of these bird species we created two groups based on two parameter values for minimum habitat patch area, 5 and 100 ha, respectively. Following the European Commission's protection targets (European Commission 2020) and empirical evidence (e.g., Hanski 2011), we also applied minimum habitat availability thresholds at the landscape level of 10 % and 30%, respectively. This matches approximately the mean landscape scale habitat proportions of 10 to 50% (median ca. 20%) of these bird species (Table S1), and the quantitative targets applied or proposed for forest conservation (Roberge 2018, Table S2).

The maps produced with such models can effectively describe the patterns of ecological qualities across forest landscapes, help set spatial conservation priorities, and improve management plans and planning practices (Lõhmus et al. 2020). Sharing maps of functional connectivity based on knowledge about birds as focal species with spatial planners can also enhance the adoption of habitat suitability index modelling and thus support landscape planning.

Table S1. Bird species preferring different tree species groups deemed potentially affected by forestry focusing on wood production, having lower or higher habitat patch size demands as well as landscape scale requirements, and which are red-listed in Sweden, listed in appendix 1 of the EU Birds Directive, and/or populations which have declined by >50% from 1980-2018 (Skogsstyrelsen 2023).

Tree species (forest type)	Bird species	Patch size requirement	Minimum landscape scale habitat proportion	Reference
Pine (ranging from open dry sparse woodland to closed canopy)	Woodlark (*Lullula arborea*)	5 ha	20%	Angelstam et al. (2004); Bosco et al. (2021)
	Nightjar (*Caprimulgus europaeus*)	50 ha	~30%	Angelstam et al. (2004); Sharps et al. (2015)
	Capercaillie (*Tetrao urogallus*)	220 ha	50% in 300 ha for lek<br>30% in a large area for lek<br>34% in 1 600 ha for lek	Sirkiä et al. (2011)<br>Wegge and Rolstad (1986)<br>Angelstam (2004)
	Black woodpecker (*Dryocopus martius*)	300 ha	20%	Angelstam et al. (2004); Nielsen et al. (2024)
Spruce (older and old-growth forests)	Willow tit (*Poecile montanus*)	10-15 ha	NA	Siffczyk et al. (2003)
	Hazel grouse (*Tetrastes bonasia*)	20-25 ha	20% in 100 ha	Åberg et al. (1995); Jansson et al. (2004)
	Three-toed woodpecker (*Picoides tridactylus)*	100 ha	10%	Bütler et al. (2004 a, b)
	Siberian jay (*Perisoreus infaustus*)	50 ha	25% in 200 ha landscape	Angelstam et al. (2004)
	Siberian tit (*Poecile cinctus*)	50-200 ha	NA	Virkkala and Liehu (1990)
Deciduous (older and old-growth forests)	Marsh tit (*Poecile palustris*)	5 ha	NA	Broughton et al. (2006, 2013)
	Long-tailed tit (*Aegithalos caudatus*)	10 ha	15%	Jansson and Angelstam (1999)
	Lesser spotted woodpecker (*Dryobates minor*)	40 ha	NA	Wiktander et al. (1992, 2001); Kost and Olsson (2025)
	Grey-headed woodpecker (*Picus canus*)	200 ha	NA	Angelstam et al. (2004)
	White-backed woodpecker (*Dendrocopos leucotos*)	100 ha	Ca. 15%	Aulén (1988); Carlson (2000)
Broadleaf (old-growth broadleaf deciduous forests and woodlands with old trees)	Red-breasted flycatcher (*Ficedula parva*)	8-10 ha	NA	Brazaitis and Angelstam (2004); Mitrus et al. (2006), Brazaitis (2011 and pers. comm.)
	Middle-spotted woodpecker (*Dendrocopos medius*)	20 ha	15%	Pettersson (1985)

Table S2. Quantitative targets applied or proposed for biodiversity conservation (updated from Roberge 2018).

Context	Target	Use	Reference
National gap analysis for forest protection in Sweden	>20% for each representative original habitat	Quantitative foundation for conservation planning	Angelstam and Andersson (2001)
Evaluation of set-aside areas for conservation in Västerbotten county	>30% protection in set-aside tracts	Recommendation for practical conservation	Uppsäll (2012)
Compilation of conservation background for area protection	>15-20% of local high conservation areas	Recommendation for practical conservation	Appelqvist (2005)
Landscape analysis for conservation core areas	>20% core areas in local landscape	Quantitative foundation for conservation planning	Bovin m.fl. (2017)
International conservation target	17%	International conservation under CBD	CBD (2010)
International conservation target for protection and management	30% of which one third strict protection	Biodiversity conservation in the European Union	European Commission (2020)
International conservation target for protection and management	30% with protection as priority	International conservation under CBD	CBD (2022)

References

Åberg, J., J. E. Swenson, and P. Angelstam. 2003. The habitat requirements of hazel grouse (Bonasa bonasia) in managed boreal forest and applicability of forest stand descriptions as a tool to identify suitable patches. Forest Ecology and Management 175(1-3): 437-444. https://doi.org/10.1016/S0378-1127(02)00144-5

Angelstam, P. 2004. Habitat thresholds and effects of forest landscape change on the distribution and abundance of black grouse and capercaillie. Ecological Bulletins 51: 173-187. https://www.jstor.org/stable/20113307

Angelstam, P., and L. Andersson. 2001. Estimates of the needs for forest reserves in Sweden. Scandinavian Journal of Forest Research Supplement No. 3: 38-51. https://doi.org/10.1080/028275801300090582

Angelstam. P., M. Manton, M. Green, B.-G. Jonsson, G. Mikusiński, J. Svensson, and F. M. Sabatini 2020. Sweden does not meet agreed national and international forest biodiversity targets: a call for adaptive landscape planning. Landscape and Urban Planning 202: 103838. https://doi.org/10.1016/j.landurbplan.2020.103838

Angelstam, P., J.-M. Roberge, A. Lõhmus, M. Bergmanis, G. Brazaitis, M. Dönz- Breuss, L. Edenius, Z. Kosiński et al. 2004. Habitat modelling as a tool for landscape-scale conservation – a review of parameters for focal forest birds. Ecological Bulletins 51: 427-453. https://www.jstor.org/stable/20113327

Appelqvist, T. 2005. Naturvårdsbiologisk forskning: underlag för områdesskydd i skogslandskapet. Rapport 5452, Naturvårdsverket.

Aulén, G. 1988. Ecology and distribution history of the white-backed woodpecker Dendrocopos leucotos in Sweden. Swedish University of Agricultural Sciences, Dept. of Wildlife Ecology. Report 14.

Bosco, L., S.A. Cushman, H.Y. Wan, K.A. Zeller, R. Arlettaz, and A. Jacot. 2021. Fragmentation effects on woodlark habitat selection depend on habitat amount and spatial scale. Animal Conservation 24(1): 84-94. doi: 10.1111/acv.12604

Bovin, M., E. Elcim, and S. Wennberg. 2017. Landskapsanalys av skogliga värdekärnor i boreal region. Metria AB på uppdrag av Naturvårdsverket.

Brazaitis, G. 2011. Forest interior species red-breasted flycatcher Ficedula parva habitat selection and conservation in intensive management areas. In: Proceedings of the fifth International Scientific Conference, Rural Development, Kaunas, Lithuania (pp. 24-25).

Brazaitis, G., and P. Angelstam. 2004. Influence of edges between old deciduous forest and clearcuts on the abundance of passerine hole-nesting birds in Lithuania. Ecological Bulletins 51: 209-217. DOI:10.2307/20113309

Broughton, R. K., S. A. Hinsley, P. E. Bellamy, R. A. Hill, and P. Rothery. 2006. Marsh Tit *Poecile palustris* territories in a British broad-leaved wood. Ibis 148(4): 744-752. https://doi.org/10.1111/j.1474-919X.2006.00583.x

Broughton, R. K., R. A. Hill and S. A. Hinsley. 2013. Relationships between patterns of habitat cover and the historical distribution of the Marsh Tit, Willow Tit and Lesser Spotted Woodpecker in Britain. Ecological Informatics 14: 25-30. https://doi.org/10.1016/j.ecoinf.2012.11.012

Bütler, R., P. Angelstam, P. Ekelund, and R. Schlaepfer. 2004a. Dead wood threshold values for the three-toed woodpecker in boreal and sub-Alpine forest. Biological Conservation 119: 305-318. https://doi.org/10.1016/j.biocon.2003.11.014

Bütler, R., P. Angelstam, and R. Schlaepfer. 2004b. Quantitative snag targets for the three-toed woodpecker *Picoides tridactylus*. Ecological Bulletins 51: 219-232. https://www.jstor.org/stable/20113310

Carlson, A. 2000. The effect of habitat loss on a deciduous forest specialist species: the white-backed woodpecker (*Dendrocopos leucotos*). Forest Ecology and Management 131: 215-221. https://doi.org/10.1016/S0378-1127(99)00215-7

CBD (Convention on Biological Diversity). 2010. Aichi biodiversity targets. https://www.cbd.int/sp/targets/

CBD (Convention on Biological Diversity). 2022. Kunming-Montreal Global biodiversity framework. In Conference of the Parties to the Convention on Biological Diversity (CBD): Montreal, Canada, p 14.

European Commission. 2020. EU Biodiversity Strategy for 2030: Bringing nature back into our lives, Communication from the commission to the European parliament, the council, the European economic and social committee and the committee of the regions. European Commission, Brussels.

Fraixedas, S., A. Lindén, M. Piha, M. Cabeza, R. Gregory, and A. Lehikoinen. 2020. A state-of-the-art review on birds as indicators of biodiversity: Advances, challenges, and future directions. Ecological Indicators 118: 106728. doi: 10.1016/j.ecolind.2020.106728

Gregory, R. D., A. Van Strien, P. Vorisek, A. W. Gmelig Meyling, D. G. Noble, R. P. Foppen, and D. W. Gibbons. 2005. Developing indicators for European birds. Philosophical Transactions of the Royal Society B: Biological Sciences 360 (1454): 269-288. https://doi.org/10.1098/rstb.2004.1602

Hanski, I. 2011. Habitat loss, the dynamics of biodiversity, and a perspective on conservation. AMBIO 40: 248-255, doi:10.1007/s13280-011-0147-3.

Jansson, G., and P. Angelstam. 1999. Threshold levels of habitat composition for the presence of the long-tailed tit (*Aegithalos caudatus*) in a boreal landscape. Landscape Ecology 14: 283-290. https://doi.org/10.1023/A:1008085902053

Jansson, G., P. Angelstam, J. Åberg, and J.E. Swenson. 2004. Management targets for the conservation of hazel grouse in boreal landscapes. Ecological Bulletins 51: 259-264. https://www.jstor.org/stable/20113314

Jansson, G., and L. Saari. 1999. Suitable habitat distribution for the long-tailed tit (Aegithalos caudatus) as indicated by the frequency of occurrence - a long-term study. Ornis Fennica 76: 115-122. https://ornisfennica.journal.fi/article/view/133506

Kost, C., and O. Olsson. 2025. Effect of habitat quality and quantity on the occurrence and persistence of the lesser spotted woodpecker (Dryobates minor) across two time periods. Biological Conservation 310: 111372. https://doi.org/10.1016/j.biocon.2025.111372

Lõhmus, A., M. Leivits, E. Pēterhofs, R. Zizas, H. Hofmanis, I. Ojaste, and P. Kurlavičius. 2017. The Capercaillie (*Tetrao urogallus*): an iconic focal species for knowledge-based integrative management and conservation of Baltic forests. Biodiversity and Conservation 26: 1-21. https://doi.org/10.1007/s10531-016-1223-6

Lõhmus, A., R. Kont, K. Runnel, M. Vaikre, and L. Remm. 2020. Habitat models of focal species can link ecology and decision-making in sustainable forest management. Forests 11(7): 721. https://doi.org/10.3390/f11070721

Manton, M.G., P. Angelstam., and G. Mikusiński. 2005. Modelling habitat suitability for deciduous forest focal species - a sensitivity analysis using different satellite land cover data. Landscape Ecology 20:827-839. https://doi.org/10.1007/s10980-005-3703-z

Mikusiński, G., and L. Edenius. 2006. Assessment of spatial functionality of old forest in Sweden as habitat for virtual species. Scandinavian Journal of Forest Research 21(S7): 73-83. https://doi.org/10.1080/14004080500487045

Mikusiński, G., E.H. Orlikowska, J.W. Bubnicki, B.G, Jonsson. and J. Svensson. 2021. Strengthening the network of high conservation value forests in boreal landscapes. Frontiers in Ecology and Evolution 8: 595730. doi.org/10.3389/fevo.2020.595730

Mitrus, C., N. Kleszko, and B. Soćko. 2006. Habitat characteristics, age, and arrival date of male Red-breasted Flycatchers Ficedula parva. Ethology Ecology & Evolution 18(1): 33-41. https://doi.org/10.1080/08927014.2006.9522724

Nielsen, A. G., A. D. Fox, and T. J. S. Balsby. 2024. Black Woodpeckers Dryocopus martius use stepping stones between woodland units. Bird Study 71(2): 143-153. https://doi.org/10.1080/00063657.2024.2329524

Öhman, K., L. Edenius, and G. Mikusiński. 2011. Optimizing spatial habitat suitability and timber revenue in long-term forest planning. Canadian Journal of Forest Research 41: 543–551. https://doi.org/10.1139/X10-23

Orlikowska, E.H., J-M. Roberge, J. Svensson, and G. Mikusiński. 2020. Hit or miss? Evaluating Natura 2000 sites for conservation of forest bird habitat in Sweden. Global Ecology and Conservation 22: e00939. https://doi.org/10.1016/j.gecco.2020.e00939

Pettersson, B. 1985. Relative importance of habitat area, isolation and quality for the occurrence of middle spotted woodpecker *Dendrocopos medius* (L.) in Sweden. Ecography 8(1): 53-58. https://www.jstor.org/stable/3682612

Roberge, J.-M. 2018. Vetenskapligt kunskapsunderlag för nyckelbiotopsinventeringen i nordvästra Sverige. Rapport 11, Skogsstyrelsen.

Roberge, J.-M., and P. Angelstam. 2006. Indicator species among resident forest birds – a cross-regional evaluation in northern Europe. Biological Conservation 130: 134-147. https://doi.org/10.1016/j.biocon.2005.12.008

Roberge, J.-M., P. Angelstam, and M.-A. Villard. 2008. Specialised woodpeckers and naturalness in hemiboreal forests – deriving quantitative targets for conservation planning. Biological Conservation 141: 997-1012. https://doi.org/10.1016/j.biocon.2008.01.010

Sharps, K., I. Henderson, G. Conway, N. Armour-Chelu, and P.M. Dolman. 2015. Home-range size and habitat use of European Nightjars Caprimulgus europaeus nesting in a complex plantation-forest landscape. Ibis 157 (2): 260-272. doi: 10.1111/ibi.12251

Siffczyk, C., L. Brotons, K. Kangas, and M. Orell. 2003. Home range size of willow tits: a response to winter habitat loss. Oecologia 136: 635-642. https://doi.org/10.1007/s00442-003-1256-x

Sirkiä, S., P. Helle, H. Lindén, A. Nikula, K. Norrdahl, P. Suorsa, and P. Valkeajärvi. 2011. Persistence of capercaillie (*Tetrao urogallus*) lekking areas depends on forest cover and fine-grain fragmentation of boreal forest landscapes. Ornis Fennica 88:14-29. http://www.ornisfennica.org/pdf/vol88-1/2Sirkia.pdf

Skogsstyrelsen. 2023. Sammanställning av fågelarter där bedömning av tillfredsställande nivå behöver göras inför skogsbruksåtgärd. Skogsstyrelsen, 2023-09-21, Diarienr 2022/1756.

Uppsäll, S. (ed.) 2012. Har vi nått målet när vi nått målet? Naturvårdsavsättningarnas roll för att uppnå miljömålet Levande skogar: exemplet Västerbottens län. Meddelande 14, Länsstyrelsen Västerbotten.

Virkkala, R., N. Leikola, H. Kujala, S. Kivinen, P. Hurskainen, S. Kuusela, J. Valkama, and R. K. Heikkinen. 2022. Developing fine-grained nationwide predictions of valuable forests

using biodiversity indicator bird species. Ecological Applications 32(2): e2505. https://doi.org/10.1002/eap.2505
Virkkala, R., and H. Liehu. 1990. Habitat selection by the Siberian Tit Parus cinctus in virgin and managed forests of northern Finland. Ornis Fennica 67(1): 1-12. https://ornisfennica.journal.fi/article/view/133294
Wegge, P., and J. Rolstad. 1986. Size and spacing of capercaillie leks in relation to social behavior and habitat. Behavioral Ecology and Sociobiology 19: 401-408. https://doi.org/10.1007/BF00300542
Wiktander, U., I.N. Nilsson, S.G. Nilsson, O. Olsson, B. Pettersson, and A. Stagen. 1992. Occurrence of the lesser spotted woodpecker Dendrocopos minor in relation to area of deciduous forest. Ornis Fennica 69: 113-118. https://ornisfennica.journal.fi/article/view/133347
Wiktander, U., O. Olsson, and S.G. Nilsson .2001. Seasonal variation in home-range size, and habitat area requirement of the lesser spotted woodpecker (*Dendrocopos minor*) in southern Sweden. Biological Conservation 100:387-395. https://doi.org/10.1016/S0006-3207(01)00045-3

Appendix S2 Tables with data behind selected graphs

Data for Fig. 3:

Table S3. Table showing the total area in ha of four different forest types, and the constituent areas of two different naturalness ranks according to Bubnicki et al. (2024), as well as the area satisfying the lowest and highest patch size (5 and 100 ha) and the functional connectivity demands (10 vs. 30%) of virtual focal species. Total area of all tree species is 21.64 mill ha.

		Total area (ha)	Rank 0.5-1.0 (ha)	Lowest species demands (ha)	Highest species demands (ha)	Rank 0.7-1.0 (ha)	Lowest species demands (ha)	Highest species demands (ha)
Scots pine								
	Mountain	379 423	281 439	279 788	53 474	249 305	247 558	38 128
	N Boreal	2 404 395	337 699	331 643	14 091	113 475	108 093	6 130
	S Boreal	3 221 981	538 534	531 704	36 072	167 074	158 307	6 223
	Hemiboreal	2 047 708	473 741	467 097	28 791	179 752	172 863	10 914
	Nemoral	138 498	16 163	14 715	0	2 049	1 324	0
	SWEDEN	8 192 005	1 647 576	1 624 947	132 428	711 655	688 145	61 395
Norway spruce	Mountain	816 084	672 318	671 439	356 644	613 672	612 851	295 195
	N Boreal	1 321 810	313 258	306 444	42 955	143 476	138 733	21 987
	S Boreal	2 488 757	557 872	551 089	37 512	224 240	215 379	20 883
	Hemiboreal	1 834 537	304 715	297 324	1 114	104 018	95 846	0
	Nemoral	222 990	19 108	17 818	0	2 671	1 929	0
	SWEDEN	6 684 178	1 867 271	1 844 114	438 225	1 088 077	1 064 738	338 065
Deciduous	Mountain	1 273 679	822 603	821 858	278 348	641 187	640 374	219 877
	N Boreal	1 597 965	166 738	158 733	0	56 105	51 103	0
	S Boreal	1 658 730	201 506	188 210	0	63 738	55 826	0
	Hemiboreal	1 395 994	267 136	258 817	115	103 030	94 001	531
	Nemoral	223 229	39 917	38 751	3 413	11 889	10 669	3 586
	SWEDEN	6 149 597	1 497 900	1 466 369	281 876	875 949	851 973	223 994
Broadleaf	Mountain	0	0	0	0	0	0	0
	N Boreal	0	0	0	0	0	0	0
	S Boreal	16 652	999	465	0	123	38	0
	Hemiboreal	409 638	92 632	82 768	4 604	42 321	36 616	3 484
	Nemoral	191 937	68 120	67 194	11 927	33 606	32 870	1 415
	SWEDEN	618 227	161 751	150 427	16 531	76 050	69 524	4 899

Data for Fig. 6, lower panel:

Table S4. Area distribution (ha) among forest owner categories in the five Swedish ecoregions of forest patches sized >5 ha for all tree species (n= 6 192 941 ha) with naturalness ranks 0.5-1.0 for all tree species merged.

	Naturalness rank 0.5-1.0; >5 ha				
	Total area (ha)	NIPF (ha)	Industry (ha)	Other (ha)	State (ha)
Mountain	2 062 813	293 592	191 084	84 186	1 493 951
N Boreal	668 730	157 436	285 354	67 475	158 465
S Boreal	1 120 157	416 365	435 437	128 602	139 753
Hemiboreal	1 060 178	662 986	98 617	202 576	95 999
Nemoral	129 430	97 228	2 238	17 467	12 497

Table S5. Area distribution (ha) among forest owner categories in the five Swedish ecoregions of forest patches sized >500 ha for all tree species (n = 780 patches) with naturalness ranks 0.5-1.0 for all tree species merged.

	Naturalness rank 0.5-1.0; >500 ha				
	Total area (ha)	NIPF (ha)	Industry (ha)	Other (ha)	State (ha)
Mountain	1 521 356	179 994	144 147	49 222	1 147 993
N Boreal	124 872	11 062	45 250	7 549	61 011
S Boreal	164 770	31 561	57 412	14 941	60 856
Hemiboreal	59 683	23 891	2 249	10 166	23 377
Nemoral	1 628	832	0	0	796

Table S6. Area distribution (ha) among forest owner categories in the five Swedish ecoregions of forest patches sized >50 000 ha for all tree species with naturalness ranks 0.5-1.0 for all tree species merged.

	Naturalness rank 0.5-1.0; >50 000 ha				
	Total area (ha)	NIPF (ha)	Industry (ha)	Other (ha)	State (ha)
Mountain	314 457	21 490	10 846	823	281 298
N Boreal	11 210	694	0	185	10 331
S Boreal	27 981	3 114	13 607	246	11 014
Hemiboreal	0	0	0	0	0
Nemoral	0	0	0	0	0
